# Pathways and Experiences of Children in Beach Vending: Findings From Cox's Bazar Sea Beach in Bangladesh

**DOI:** 10.1002/puh2.70163

**Published:** 2025-11-27

**Authors:** Md. Ziaul Islam, S. M. Sharf‐Ul‐Alam, Zannatun Naeem Keya, Miskatul Jannat, Zakia Alam, Zakia Ferdausi Khan, Arpan Maitra, Md. Abdullah Saeed Khan

**Affiliations:** ^1^ Department of Community Medicine National Institute of Preventive and Social Medicine Dhaka Bangladesh; ^2^ Department of Biomedical Engineering National Institute of Preventive and Social Medicine Dhaka Bangladesh

**Keywords:** Bangladesh, beach vending, children, Cox's Bazar, experiences, pathways

## Abstract

**Background:**

Child beach vendors comprise a unique segment of child laborers, characterized by distinct characteristics, experiences, and challenges. Despite being vital players in the economy of Cox's Bazar sea beach, these underprivileged children remain overlooked in policy planning and welfare programs due to the mobile, seasonal, and informal nature of work. The challenges and realities confronted by the child beach vendors remain less explored. This study aimed to investigate the pathways leading children to beach vending and highlight their relevant experiences.

**Methods:**

This phenomenological type of qualitative study was conducted from September to December 2023. The study enrolled 18 child beach vendors conveniently from the Kolatoli, Sugondha, Laboni, and Patuartek points of Cox's Bazar sea beach. We obtained informed assent from each child, as well as consent from their guardian, and collected data through in‐depth interviews using an interview guide.

**Results:**

A total of 6 themes and 18 sub‐themes emerged through phenomenological reduction. Three themes describing pathways to beach vending were identified: economic (poor family income, loss of earning members, and escalated family expenses), psychosocial (social negligence, humiliation at school, and motivation), and environmental (imitating peers, vending opportunities at the beach, and family environment). Three themes emerging from the experiences of child vendors included physical challenges (tiredness, illness, and injuries), deprivation and coping strategies (interrupted education, school experience, and grouping), and psychological impacts (mental stress, satisfaction, and dreams).

**Conclusion:**

Engagement of children in beach vending is a violation of labor law and children's rights. Poverty, unmet basic needs, and social negligence were major push factors, whereas earning opportunity was the primary pull factor for child beach vending. Child vendors were found to experience detrimental health consequences, academic disruptions, and various forms of abuse. Coordinated interventions, including school‐based incentives, vocational training, and social safety net programs, could reduce both the necessity and appeal for children to become beach vendors.

## Graphical abstract

The graphical abstract illustrates the pathways driving children into beach vending—economic hardship, psychosocial influences, and environmental factors—and their lived experiences, including physical challenges, deprivation, and psychological impacts. It visually highlights the push‐pull dynamics and underscores the need for targeted interventions to protect and rehabilitate child beach vendors in Cox's Bazar.

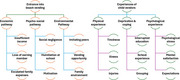

## 1 Introduction

Exploitation and abuse of children in various laborious and hazardous activities are quite common worldwide. Despite the expansion of education and the enactment of labor laws, child labor persists at an alarming rate in Asia and Africa, with child employment rates of 61% and 41%, respectively [1]. More than 4 million children in India work for more than 6 hours per day, and an additional 2 million work seasonally [2]. According to age‐appropriate working hours defined by the SDG, approximately 6.8% of children in Bangladesh engage in laborious activities, with over 1 million in hazardous work [3]. Far from decreasing, this percentage has increased from 8.7% to 8.90% during the period from 2012 to 2022 [4].

According to the International Labor Organization (ILO), child labor refers to any work that robs children of their childhood, potential, and dignity and is harmful to their physical and psychological development [5]. Globally, around 150 million children are engaged in child labor [6, 7, 8], and among them, child vendors constitute a substantial segment [9]. Child vendors are most pervasive on streets, highways, road intersections, and places of public gatherings, selling a wide range of products. Despite enjoying a certain level of autonomy, these children often work long hours for minimal wages, facing abuse [10] and unstable employment opportunities dictated by chance and a restrictive social structure that hinders their purposeful entrepreneurial efforts [11]. Street vending has both positive and negative impacts on the informal economy of the developing world. Reducing urban unemployment and ensuring affordable goods for the low‐income urban population are positive outcomes. At the same time, road congestion, dirt, tax evasion, and an increased risk of criminal activities are negative consequences of street vending [12]. In Bangladesh, approximately 250,000 street vendors operate in Dhaka city alone, and their numbers are constantly increasing across the country, including at Cox's Bazar, the country's prime tourist hub [13].

Cox's Bazar, a southeastern district in Bangladesh, offers a unique 120 km long, unbroken sandy sea beach with various tourist destinations. Many locals, including children, earn their livelihood by vending various products and services to tourists at popular beach points. They contribute significantly to sea‐beach‐based tourism activities and the informal economy [14, 15]. A 2015 survey reported that 771 children were engaged in vending and other activities on the beaches of Cox's Bazar [16], though the exact number is unknown due to the mobile, seasonal, and informal nature of their work [13]. Child beach vendors usually operate within the beach vicinity and exhibit distinct characteristics, experiences, and challenges. These children frequently walk on foot to sell their products, carrying plastic sheets, cloths, baskets, or open pushcarts to ensure easy and full display [17]. They vend a wide range of products, including snacks, drinks, souvenirs, toys, handmade accessories, and crafts, whereas others work as tourist guides, horse riders, bike drivers, photographers, and more [18]. Many of them craft innovative and culturally distinctive products, typically unavailable elsewhere, which draw significant tourist attraction [13].

However, child beach vendors face income uncertainties due to seasonal fluctuations influenced by weather, holidays, and festivals [19, 20, 21, 22], indicating child vendors must capitalize on peak days regardless of health or schooling needs. Adversities of political tensions, wars, and pandemics may further constrain their income [23]. They face unique occupational hazards, like exposure to intense sunlight, scorching sand, rain, heavy carriage, constant movement, long working hours without holidays or weekends, and detrimental impacts on their physical and psychological health. Moreover, these beach vendors, working outside the formal economy and regulatory frameworks, often remain overlooked in policy planning and welfare programs [24]. Unlike their counterparts in the formal economy, these children are also excluded from social security benefits like medical care and labor protections [13].

Common health problems faced by child vendors include exhaustion, pain, acute infections, injury, and exposure to pollutants [25]. Insufficient calorie intake and excessive physical labor lead to malnutrition among these children, resulting in impaired physical growth, reduced academic performance, and diminished earning potential—consequences that cannot be fully compensated by nutritional interventions in later stages of life [26]. Moreover, they frequently experience insecurity, discrimination, forced labor, theft, exploitation, harassment, sexual assault, and human trafficking [27, 28, 29, 30, 31]. Such maltreatment and neglect have a profound long‐term impact on children, including impairment of brain and psychological development, erosion of self‐esteem, reduced productivity, potentiality, and educational proficiency, and a higher propensity to engage in risk behaviors like criminal activities and drug abuse—each carrying a high societal cost [16, 32]. Studies in Bangladesh show 60% of street vendors suffer from anxiety due to eviction fears and social neglect [23].

The UN Charter on Children's Rights advocates protecting children from harmful jobs that threaten their education and development [33]. However, the socioeconomic challenges in low‐resource countries force marginalized families to involve children in income generation [34]. Many of these children have to participate in informal economic activities as survival strategies for their families [35], often sacrificing education [36]. Research in Nigeria found that 60.5% of child vendors dropped out of school. Despite aspiring to higher education, most couldn't achieve their dreams while generating only 25% of the family income. Nearly one‐third of child vendors are robbed of their earnings, and 6% of girl vendors face sexual harassment [30].

According to UNICEF, these beach vendors can be classified as street children, as they spend a significant portion of their day at the beach to earn a livelihood [16]. Unveiling the pathways leading children into vending is crucial for addressing and curbing the prevalence of child vendors. Understanding the child vendors’ experiences can provide valuable insights for policymakers to implement protective measures against child labor and provide educational opportunities. However, very few studies have explored the lived experiences and challenges faced by child beach vendors in Bangladesh. Hence, this study aimed to explore the pathways leading children to beach vending and their multifaceted experiences.

## 2 Methods

### 2.1 Study Design and Settings

**FIGURE 1:**
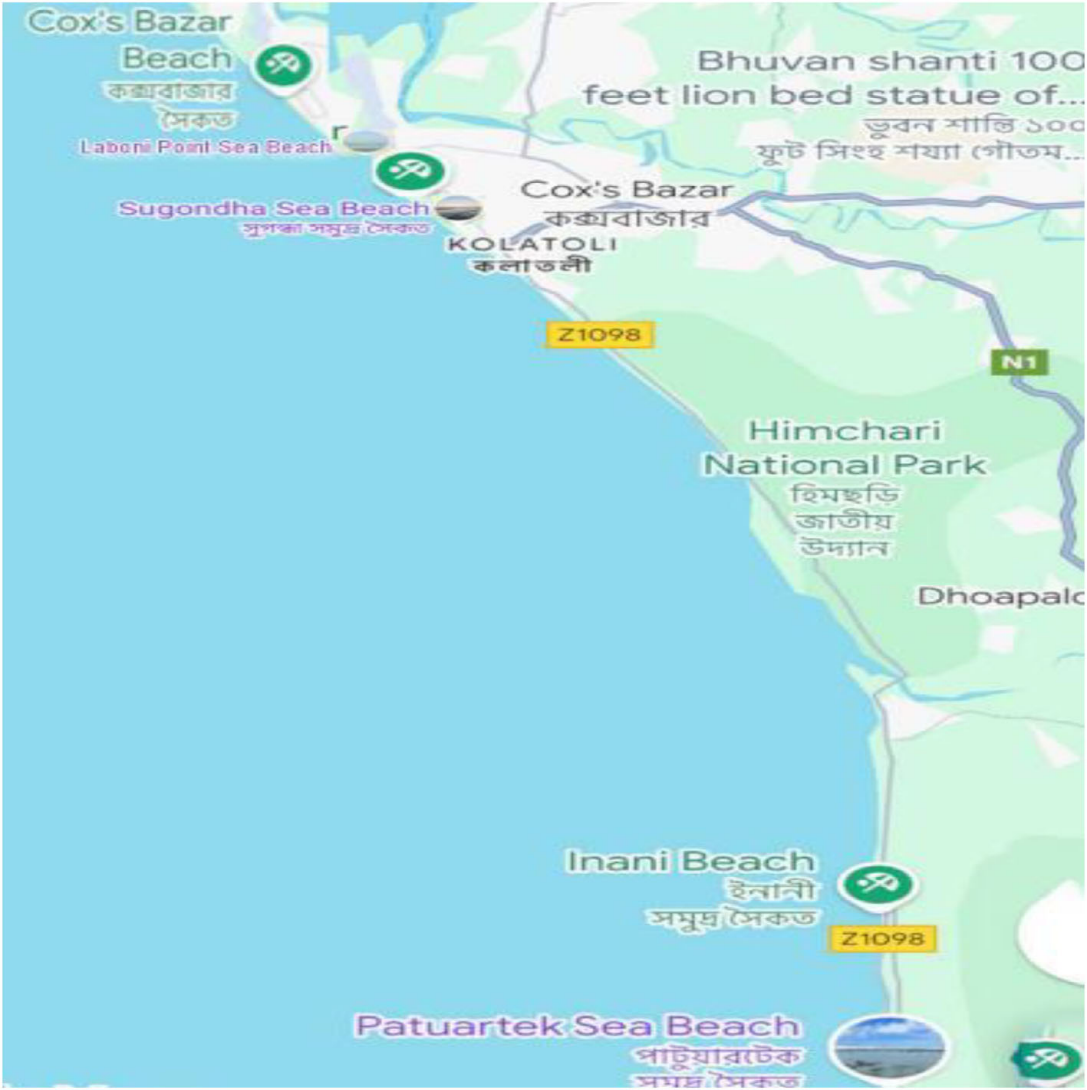
Map showing study areas. Map data: Google, Maxar Technologies; Accessed on 4 October 2025

We conducted a phenomenological qualitative study among child beach vendors between September and December 2023 at Cox's Bazar sea beach in Bangladesh. The study focused on four frequently visited areas on the beach: Kolatoli, Sugondha, Laboni, and Patuartek (Figure 1).

### 2.2 Study Population and Sampling

The study enrolled 18 child beach vendors aged 10–15 years who were permanent residents of the catchment areas of the study places through convenience sampling. Data enumerators approached vendors meeting age criteria, explained study objectives, and arranged house visits for data collection.

### 2.3 Data Collection

Enumerators verified participants’ age as well as nationality through birth certificates or vaccine cards. Informed written assent from children and consent from parents were obtained. In‐depth interviews (IDIs) using an interview guide (Supplementary File) were conducted in Bangla with simple, child‐friendly language, lasting 40–50 min. Interviews began with background questions before exploring pathways to vending and experiences. First‐person observations were made, and field notes were taken on the activities of the child vendor before approaching for IDI. This information supplemented the recorded interviews for data triangulation. Each audio record was transcribed verbatim by the enumerators.

### 2.4 Data Analysis

We employed descriptive phenomenological analysis following these steps [37]:

*Epoche/Bracketing:* Researchers set aside preconceptions to obtain unbiased participant opinions.

*Phenomenological reduction:* This included horizontalizing (listing relevant data and coding similar information), constructing sub‐themes (arranging similar data into meaningful units), and thematic clustering (economic, psychosocial, and environmental themes under pathways; and physical experiences, deprivation and coping, and psychological experiences under experiences of beach vending domain).

*Construction of composite structural descriptions:* Narratives were structured on the basis of themes, incorporating all participant responses.

### 2.5 Ethical Clearance

We obtained the ethical approval from the Institutional Review Board of the National Institute of Preventive and Social Medicine (Ref: NIPSOM/IRB/2023/08 Date: 12.01.2023). Permission was taken from the administrative authority to approach child vendors in the beach area for study purposes. Informed assent from participants and consent from parents were obtained before they participated in the study. All study procedures were conducted following the latest guidelines of the Declaration of Helsinki.

## 3 Results

### 3.1 Background Characteristics of Children

The majority (12) of the participants were males and belonged to nuclear families. Most (16) lived in kacha houses (made with nondurable materials like mud, thatch, bamboo, and leaves). Some participants (5) had single parents, and 3 were major wage earners in their families. Day laborer was the most common (7) paternal occupation. Female vendors primarily sold flower garlands and ornaments, whereas male vendors sold water bottles, stone ornaments, seasonal fruits, and tea or offered services like horse riding and photography. Half of the participants (9) earned up to 400 BDT daily on average, whereas 8 earned up to 600 BDT. Only one participant reported an average daily income exceeding 600 BDT. A few participants did not attend school at all, whereas some (11) were either currently at or had completed the primary level of education (Table 1).

**TABLE 1:** Background characteristics of the participants.

Attributes	Findings
Age group	10–15 years, mean −13.2 years
Sex	Male 12; female 6
Family type	Nuclear 12; joint 6
Family structure	Single‐parent family (death/abandonment of father) 6; two parent family 12
Father's occupation (*n* = 13)	Day labor 7; fisherman 3; unemployed 2
Main wage earner of the family	Father 10; mother 3; elder sibling 2; child vendor h/herself 3
Housing condition	Semi‐pucca 2; kacha 16
Education (current level)	No formal schooling 2; primary level 11; secondary level 5
Products/Services vended	Flower garlands 4; stone ornaments 4; water bottles 4; seasonal fruits 2; tea 2; horse riding 1; photography 1
Average daily income in the last 2 months (BDT)	Up to 200: 4; 201–400: 5; 401–600: 8; >600: 1

### 3.2 Pathways to Beach Vending

**FIGURE 2:**
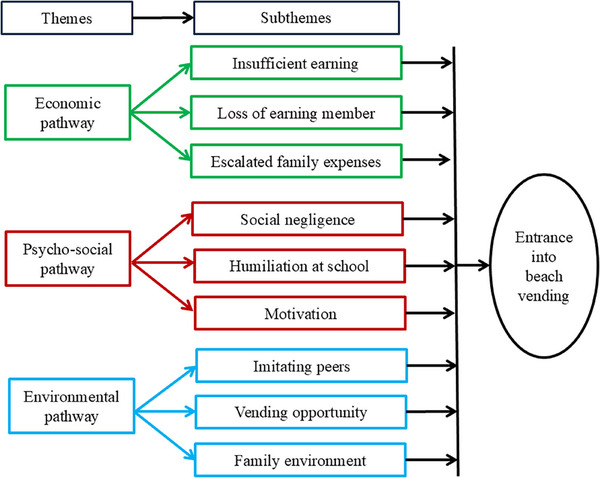
Pathways driving children to beach vending.

Three distinct pathways into beach vending have emerged: economic, psychosocial, and environmental. The economic pathway reflects families’ harsh financial realities—insufficient income and unmet basic needs pushing children to beach vending. The psychosocial pathway incorporates children's thoughts, emotions, and motivations shaped by social neglect, isolation, and family expectations. The environmental pathway highlights contextual factors like family setting, peer influence, and tourist opportunities that make vending an attractive option to earn (Figure 2).

### 3.3 Economic Pathways

#### 3.3.1 Insufficient Earning

Participants reported that poor family income didn't cover their basic needs. Despite parents’ hard work, earnings were inadequate. As Participant 1 stated the following:

“My father cuts wood from the nearby jungle and sells it in the local market. He is getting old, and he can't collect enough wood as it is very tough and laborious work. Occasionally, he also works as a day laborer, but his income is barely enough to feed us.”

Some mentioned receiving unfair prices for their products. Participant 2 explained the following:

“My father catches fish day and night in the sea and sells them in the local market. But he doesn't get the proper price. Although fish are sold at a high price in the market, the middlemen who buy fish from my father offer a much lower price. Hence, we don't earn enough money to maintain our family.”

Most of the participants mentioned that their family income is barely sufficient to afford three full meals a day. Participants 1, 2, 5, and 8 mentioned that their parents search for work every day. Some days they find it, and some days they don't. Many participants appeared malnourished, reflecting poor calorie intake, which reflects their poor economic background.

#### 3.3.2 Loss of the Earning Member of the Family

The death or departure of the main earners of the family devastated families. Some participants mentioned the loss or abandonment of their fathers, which left their mothers struggling to maintain the household. However, gender‐based wage discrimination made mothers’ earning potential insufficient despite equal work effort. Participant 15 described the following:

“In the absence of a fatherly figure, at the age of 8, I had to assume the role of the guardian and the bread earner for my family. Though it is very tough, I have to do it.”

Participant 13 shared that his father's paralysis forced his mother and siblings into income‐generating work regardless of age.

#### 3.3.3 Escalated Family Expenses

Sudden increases in family expenses compelled some children to play earning roles. Medical treatment costs frequently left the families impoverished or forbade them from seeking treatment for health problems. Participant 2 discussed financial burdens from her mother's abdominal tumor treatment.

Dowry presented another financial burden. Participant 1's father borrowed money at a high interest rate for his sister's marriage dowry, worsening their economic situation. Participant 17 noted the following:

“My elder sister was married off at only age 15 against her will to reduce family expenses. However, my father had to give a dowry for the marriage.”

Natural disasters destroyed homes and property, which created additional financial strain. Most participants lived in vulnerable structures requiring frequent repairs. Some participants complained of loss of homes and belongings due to natural calamities, including storms and heavy rainfall.

### 3.4 Psychosocial Pathways

#### 3.4.1 Social Negligence

Participants disclosed exclusion from social events due to their socioeconomic status. Participant 14 noted a changing attitude of neighbors after his father's death:

“When my father was alive, our family was well‐respected and evaluated by our neighbors. During those days, when I woke up in the morning, my mother affectionately prepared me for school after feeding me breakfast and a warm cup of tea. But with the death of my father, our financial condition deteriorated… The attitude of our neighbors has also changed towards us.”

They faced difficulties accessing public representatives and officials compared to higher social classes. Participant 4 reported that, following her father's death, some of their property was seized by relatives and had not been recovered, despite complaints made to the local political representative. Participants mentioned reluctance to attend social gatherings due to the cost of gifts and being denied loans even in emergencies.

#### 3.4.2 Humiliation at School

Some participants faced harsh treatment from teachers for lacking proper school uniforms and educational materials. Participants 2, 7, and 15 experienced bullying and discrimination from classmates who viewed them as inferior.

#### 3.4.3 Motivation

Child vendors exposed a remarkable awareness of economic hardships in their families. Most expressed strong desires for financial contributions to lessen parents’ burden. A few participants mentioned that their parents highly appreciated their decision to get involved in earning. Participant 12 worked to support his younger sister's education. Participant 1 stated confidently the following:

“Women have to take part in every affair to support the family when men can't fulfil all needs. We must maintain the family by doing household and external work.”

This self‐realization regarding contribution to family income motivated some of the participants to work in such harsh conditions. However, a few were forced to work even against their will as a survival strategy for themselves as well as their families. Participant (17) shared the following:

“My father told me to stop schooling and work from dawn to dusk. But I want to be a graduate from a university, I told him. However, he argued that education cannot be received on an empty stomach.”

### 3.5 Environmental Pathways

#### 3.5.1 Imitating Peers

Many children were influenced by friends, neighbors, or family members to engage in beach vending. Participant 16 explained the following:

“People like us have to do some work to earn and support the family. See, children (pointing to a young boy of 7–8 years), younger than me, are also doing such work. The boy had to discontinue his studies after his father's death. He has now resumed his studies with his earnings.”

Participant 4 began vending after seeing her elder sister's experience:

“I saw my elder sister earning money by selling a flower crown and giving it to my father till she got married. For her earnings, she enjoyed the opportunity to participate in family matters, and her opinions were considered with importance.”

Some of the children initially accompanied vendor friends before starting their vending activities.

#### 3.5.2 Vending Opportunity at the Beach

Participants lived close to tourist points, making beach vending convenient for them. Some participants said that preparing their vending products required minimal skill and cost. A few participants mentioned that family members such as mothers and sisters help to collect or manufacture the products. During peak seasons, they earned between 300 and 1000 BDT ($2.75–9.17), though income dropped significantly during off‐seasons and natural disasters. However, some participants mentioned that tourist flow has increased throughout the year, even during the off‐season.

#### 3.5.3 Family Environment

Constant scarcities and insufficient utilities create poor family environments marked by restlessness, unhealthy competitiveness, and even toxic interaction among members, propelling some children into vending. Participant 5 mentioned traveling long distances for drinking water. Some families lacked electricity and relied on solar panels with limited capacity to meet the needs of all members at all times. Female children sometimes felt like burdens to their families. Participant 1 shared the following:

“I remember my father used to love my elder sister and me very much. But after my younger sister's birth, he does not even talk to us properly, as we are supposed to be a burden to him.”

Some noted delinquent family members as additional motivations for earning. Participant 4 mentioned her drug‐addicted brother, who threatened the family for money.

### 3.6 Experiences in Beach Vending

**FIGURE 3:**
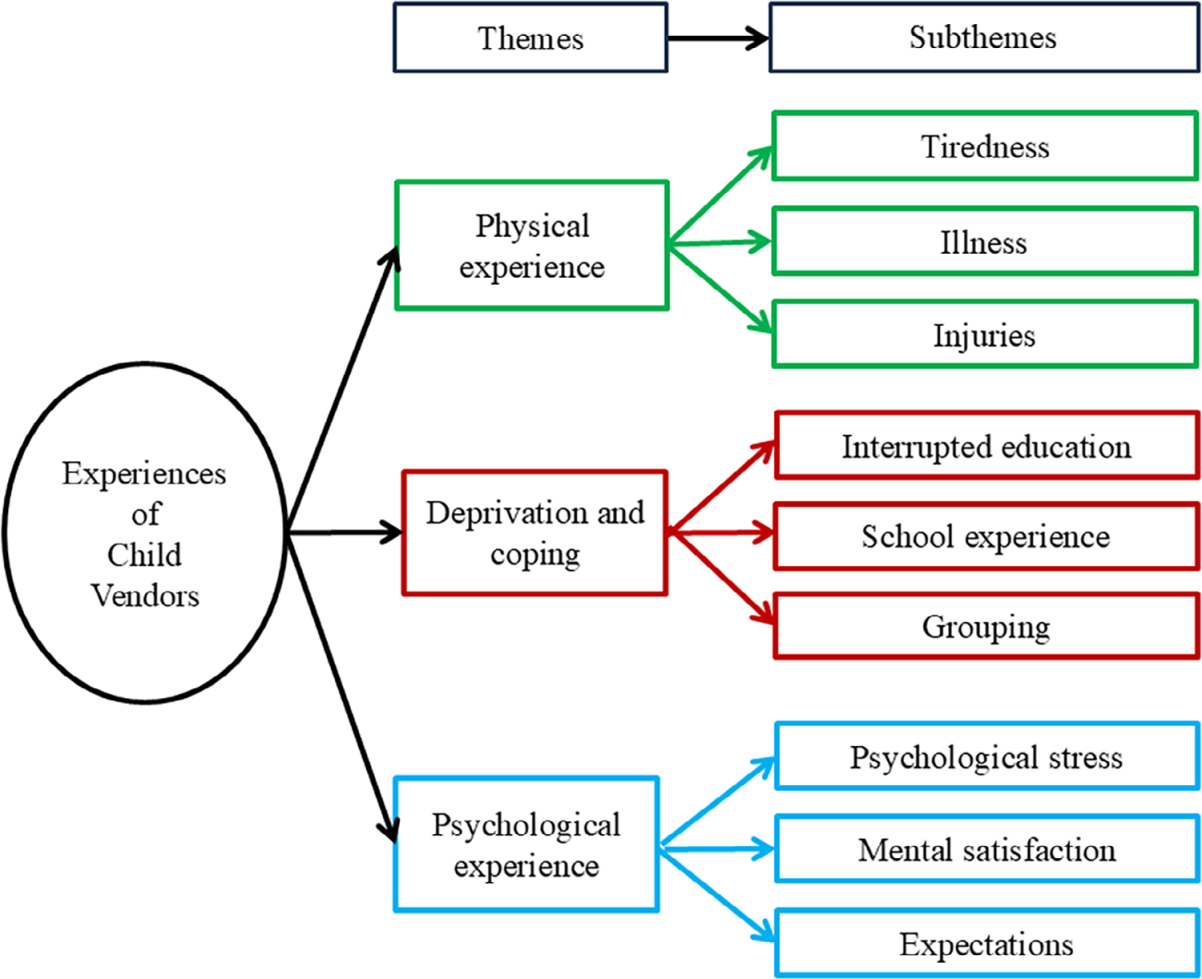
Experiences of children in beach vending.

Under experiences in the beach vending domain, three major themes emerged: physical experiences, psychological experiences, and deprivation and coping. Physical experiences addressed body strains and physical health‐related problems of children associated with vending. Psychological experiences incorporated emotional and mental consequences like stress, internal conflict, and aspirations experienced by child vendors. Deprivation and coping reflect the loss of essential childhood rights such as education, joy, and security, along with the strategies children adopt to overcome these hardships (Figure 3).

### 3.7 Physical Experiences

#### 3.7.1 Tiredness

Children worked long hours in hot, humid beach conditions, leaving them exhausted. The working hours varied among participants, ranging from 4 to 16 h per day, depending on earning opportunities and financial crises. Some participants began working from early morning and continued till night, whereas others worked a few hours in the afternoon. Participant 5 explained the following:

“I have to work 8 hours daily and seven days a week to earn food for my family. I feel too tired, and I cannot enjoy a holiday even for sickness. If I take a rest, my family may have to go without food on that day.”

Participant 11, a horse rider, described constant thirst and fatigue during work. Sometimes he could not drink enough water, as he had to purchase it when free sources were unavailable. Some participants were found sweating profusely and too tired, especially in the noon and afternoon.

#### 3.7.2 Illness

Common health issues included weakness, exhaustion, dehydration, muscle aches, colds, and skin problems. Participant 11 described the following:

“Every night, I feel severe muscle pain and stiffness during sleep. My body remains so weak that I feel dead and often find it very difficult to get up from bed the next morning.”

Participant 7 complained of large, itchy skin lesions on his arms and legs, exacerbated by limited bathing opportunities.

#### 3.7.3 Injuries

Participants reported various injuries from work activities. One sustained a severe shin injury requiring 18 stitches. Participant 11 experienced ankle sprains, soft tissue injuries, and a leg fracture. Participant 14 suffered throat pain from constant shouting to attract customers, explaining the following:

“I know the meaning of competition. If I stop or even reduce my efforts, others will grab the opportunity and attract customers. That will decrease my income, and I might even lose the opportunity to sell tea here.”

### 3.8 Deprivation and Coping

#### 3.8.1 Interrupted Education

Many participants expressed interest in education but faced obstacles due to work pressure. Participant 15 shared the following:

“I had to discontinue my studies to fulfil the family needs and educational expenses of my younger siblings. But I would like to resume my studies again. Although I know it is very tough.”

Some never attended school, whereas others worried about poor academic performance due to interruptions by vending activities. Many were encouraged to skip school during peak tourist seasons.

#### 3.8.2 School Experience

Some participants faced bullying from classmates. Participants 3 and 18 stated they have been teased about their profession. Some seasonal vendors hid their work from classmates. Those attending school faced punishment for absenteeism. November and December created dilemmas as these months coincided with both peak tourist season and final exams. Most participants preferred to discontinue or at least interrupt academic activities to earn through vending.

#### 3.8.3 Grouping

Some vendors worked alone, whereas others formed groups based on age, residence, or product type. One participant explained the following:

“We are four friends selling flower garlands and stone ornaments here in Kalatali Beach. We come together from our house, sell our products, and play together when customers are unavailable. We compete with each other for sales, but we also protect each other during any problematic situations.”

Groups provided them protection against harassment, theft, and abduction. Some mentioned paying bribes through elder vendors to secure their selling locations. However, a few participants expressed that members of the groups compete and quarrel with each other to sell their products to customers.

### 3.9 Psychological Experiences

#### 3.9.1 Psychological Stress

Some child vendors faced significant mental trauma. Family income concerns remained paramount. Domestic violence was common in impoverished households. Participant 1 described the following:

“Out of frustration, almost every day, my father beats my mother for small issues, like when she complains about the unavailability of different necessary things in our house.”

Some children faced verbal or physical abuse for not meeting sales targets. Others had to share earnings with business owners or local gangs. Participant 16 noted the following:

“For every soft copy of the picture, I charge 5 BDT, from which I have to give 2.5 BDT to the owner of this DSLR camera. I wish I had a camera of my own so that I don't have to share my hard‐earned money.”

Many worried nightly about the next day's earnings and protecting their money from theft. Some participants expressed their frustration over the harsh behavior of the tourists and the beating of the personnel from various authorities. A few participants narrated that they had to tolerate threats, insults, and humiliation from senior vendors for the selling place. Two female participants also mentioned their fear of being harassed sexually. Most of the participants reported not getting any legal support in case of harassment and abuse.

#### 3.9.2 Mental Satisfaction

Despite hardships, participants expressed satisfaction in contributing to their families. Participant 4 stated happily the following:

“I give all my money to my mother just to see her happy and smiling face.”

Participant 12 found joy in supporting his younger sister's education despite discontinuing his own. Participant 15 proudly stated the following:

“I now look after my mother and younger sister solely. I don't allow any of my family members to work, not even my mother. I don't want to see my family members going through the same difficult paths.”

This satisfaction provided mental strength to endure hardships. Participant 14 described his dedicated approach:

“I pour my heart into every cup of tea, filling them with warmth and kindness. I try to serve every cup with a radiant smile and a friendly gesture to every customer, making my tea taste even better.”

Some participants enjoy being at the beach, both for playing with fellow vendors and earning money.

#### 3.9.3 Expectations

Participants shared aspirations to become doctors, engineers, pilots, and teachers. Some participants expressed a desire to launch their own business. They dreamed of alleviating family poverty. Participant 13 stated determinedly the following:

“I promised my mother that one day I will be sitting in the ship that I will sail as a Captain. I will take her to different countries to visit.”

Some expressed desires to continue their education as well as help future generations of children to be educated. Participant 17 shared empathetically the following:

“When I am wealthy, I will help poor children to go to school so that they don't have to face harsh realities and difficulties like me.”

Participant 3 added tearfully the following:

“When I am a schoolteacher, I will never scold any children for clean dress and shoes because I know the cost of proper cleaning.”

Many hoped for government intervention to help them escape poverty.

## 4 Discussion

The present study focused on the pathways leading to beach vending and the lived experience of conveniently selected child vendors from four prominent tourist spots at the Cox's Bazar sea beach. Some participants had no choice but to engage in vending to meet basic needs essential for their families’ survival, whereas others chose it to supplement the family income and cover expenses like education. These findings align with a Pakistani study, which reported that 27% of child labor is driven by necessity, 31% by voluntary choice, and the rest by feasible earning opportunity [38]. However, all the participants of this study represent a poor and marginalized segment of Bangladeshi society struggling with very low family income and unmet basic needs. At present, tourism has expanded at Cox's Bazar and increased earning opportunities in both formal and informal sectors, but the earning potential remains limited for poor, less educated, and unskilled individuals [39]. Situation further deteriorated after the influx of millions of Rohingya refugees, disrupting the balance between the supply and demand of labor in the market [40]. The average daily wage dropped by 14% as refugees accepted much lower wages, and many low‐skilled local workers were displaced from agriculture and fisheries, forcing them to engage in riskier and unstable livelihoods [41]. Moreover, the entry of Rohingyas in small business and entrepreneurial activities like tea stalls, rickshaw pulling, and vending negatively affects the locals engaged in similar professions [40]. In this context, child vending emerges as a coping strategy for families, demanding minimal investment and skills, offering flexible working hours, and supplementing family income.

Most of our participants stated that poverty was the main factor driving them to engage in vending, a finding supported by evidence from other parts of the world [9, 42]. However, poverty and child vending have a bidirectional relationship, with the vending of children being both the cause and consequence of poverty [43]. Poverty creates a vicious cycle for these children, resulting in malnutrition, physical weakness, and illness, which, in turn, diminishes their working capacity and intensifies their economic hardship [44]. Child labor patterns often pass generationally [45], where vendor parents may involve children in their work, exposing them to harsh external environments [35]. This creates an unskilled workforce, negatively impacting national development [46]. Moreover, poverty permanently harms children's development, depriving them of nutrition, health services, sanitation, education, and other rights [47].

Family conditions and composition significantly influenced children's entry into vending, especially for orphans and children from single‐parent or child‐headed families, who bear the responsibility of caring for other members, including those with disabilities. These findings align with similar previous studies [9, 48], highlighting that underprivileged children often face various deficiencies and work to overcome them [19]. Moreover, families with unmet basic needs often send their children to work, including risky jobs, contributing to 50% of primary school dropouts in Bangladesh [49]. Additionally, a gendered division of child labor exists, primarily influenced by sociocultural factors. Girls are typically assigned domestic tasks and, in some contexts, street vending, whereas boys are more often sent to work outside, such as in factories and on the streets, particularly in developing countries [10, 50, 51, 52]. Naz et al. reported that cultural norms, joint family structure, agriculture‐based society, financial debt, and family disharmony promote child labor, demanding an incentive‐based education system, strict child protection, labor law enforcement, and saving schemes for the families to minimize the problem [53].

Education remains unattainable for some of our participants, and those who are enrolled in school must work around class hours, causing significant physical and mental strain. Parents often encourage school absence for work, and many develop truancy [54]. Some of our participants got help from their mothers in preparing goods for vending. Although parents may understand the importance of education, they struggle to afford food, let alone educational expenses [55]. However, parental education positively influences children's education and reduces engagement in unskilled jobs [24]. On the other hand, poor school environments and high education costs discourage schooling of children [10]. According to the Constitution of Bangladesh and the National Education Policy 2010, education is recognized as a basic human right, and primary education is mandatory. Government primary and secondary schools do not charge any tuition fees and provide free textbooks as well as stipends for students. However, families still have to bear the expenses of other educational materials, non‐tuition fees, transportation, and sometimes food costs during school hours. The stipend provided is barely sufficient to cover these costs [56]. Evidence from India and Pakistan suggests that reducing schooling‐related costs can decrease the probability of child employment [57, 58].

The child vendors in our study face various forms of exploitation, extortion, and harassment. Those working under employers face additional pressure to meet sales targets. Children working in the informal economy are particularly vulnerable to abuse as they perform various jobs under different employers [59]. To cope, children form informal networks for protection, make agreements with authorities, and sometimes limit the number of vendors to reduce competition [60]. Other strategies they follow include night vending [61] and unethical means of bribing authorities [62].

Our study found that some young vendors face an overwhelming struggle to balance long, exhausting work, supporting their families, and pursuing their education. Such immense weight of responsibility at childhood can lead to long‐term physical and psychological trauma, making them vulnerable to various diseases [10]. Moreover, occupational conditions may affect their health, leading to hearing problems, headaches, skin diseases, infections, and injuries [23]. Child laborers in the dried fish sector of Cox's Bazar were found to face similar hardships [63]. According to WHO, excess work, lack of control over working conditions, low wages, lack of family and recreation time, difficulty in managing school time, physical punishment, isolation, and subjugation have grave consequences on the psychological health of child vendors [43]. In addition, mistreatment, bullying, exploitation, social neglect, and breakdown of social networks and emotional bonds are also common among vendors’ families [35, 64].

The ILO's Convention 138 sets the minimum employment age at 15 years [10]. The United Nations Convention on the Rights of the Child protects children from “all forms of physical or mental violence” (Article 25) [65]. Although Bangladesh has ratified the Labor Act 2006 and the Children Act 2013, a wide gap exists in implementation, particularly in the informal sector. The absence of a robust age verification system, easy availability of child employment, recruitment of children in informal and undocumented ways, persistent poverty, unstable adult income, and rising unemployment during the COVID‐19 pandemic continue to hinder the progress toward reducing child labor in Bangladesh [66].

Despite all odds, most children shared their expectations, primarily centered around standard earning opportunities and better living conditions for themselves and their families—similar to the findings of another study [16]. They are sacrificing their childhood for a bright future with mixed emotions of trauma and satisfaction. Their earnings, though small, still sustain these hopes—they believe better days are ahead. However, poverty overshadows their aspirations and childhood joy. Many participants aspired to continue their academic journey without interruptions.

The entry pathways for beach vending and the lived experiences of child beach vendors are not separate rather interlinked, creating a self‐perpetuating cycle, commonly observed for child labor [43]. Economic hardship, social neglect, and enabling environment in family and surroundings both propel and lure children in vending to work harder and longer periods. This results in detrimental physical and psychological outcomes, academic disruption, and various forms of exploitation. In turn, these experiences escalate poverty and deepen social marginalization, gradually normalizing vending as an acceptable livelihood for children and perpetuating it across generations.

The study's limitations include selecting participants from only four beach points, and no follow‐up or feedback was obtained. The findings could not be generalized to all child beach vendors of Cox's Bazar.

However, completely stopping child vending immediately without ensuring family financial security would be unwise. A study across several developing countries found 77% of working children prefer combining work and school, and 67% would even violate laws if prevented from working [64]. Registration of all child vendors, adjusting school hours and vending schedules, and strengthening school‐based welfare programs like food for education, stipends, and provision of academic and vocational training could protect their rights as well as economic safety. However, a study found that rural poor youth in Cambodia and Laos tend to prefer informal training courses or apprenticeships over formal national vocational programs, as these are more accessible, affordable, and flexible, while also offering hands‐on learning, direct customer interaction, and opportunities for home‐based or self‐employed work [67].

Social safety nets covering basic needs, healthcare, skill development, and job opportunities should be developed. Focusing on creating income‐generating opportunities for adults in informal sectors, allocating dedicated vending zones, and providing legal protection can reduce the prevalence and sufferings of child beach vendors [18]. Providing skills training and business support to mothers could potentially free their children from the burden of earning [68]. Various studies suggested that combined interventions, like cash transfer, behavior change communication (BCC), and food transfer, have a significant positive impact on child nutrition and health, which could be helpful to reduce child vending [69, 70, 71]. Smith argued that raising income to a certain level through microfinance would encourage parents to withdraw their children from laborious jobs [72]. However, Goulart et al. [73]demonstrated that a multifaceted and comprehensive approach is required to address the child labor problem. In addition to economic development at the family and national levels, technological advancements in the agricultural and industrial sectors also help to reduce child employment by increasing adult workers’ productivity. Additionally, family dynamics, cultural beliefs, and gender roles play a significant role in the prevalence of child labor and should be considered when developing a comprehensive strategy [73]. Social awareness programs against practices, like dowry and domestic violence, should be implemented. Relevant laws should be strictly enforced, with children's opinions to be considered in policy development.

## 5 Conclusion

Poverty was the primary driver for children to engage in beach vending, whereas earning opportunities at the beach were the motivating pull factor. Without ensuring the fulfillment of basic needs and a certain level of economic stability, the appeals for child beach vending would not dissolve. To ensure this, considering the opinions of all relevant stakeholders as well as the beach vending children, long‐term coordinated strategies and programs should be implemented.

## Author Contributions

Md. Ziaul Islam and S. M. Sharf‐Ul‐Alam conceptualized and designed the study. S. M. Sharf‐Ul‐Alam, Zannatun Naeem Keya, Miskatul Jannat, Zakia Alam, Zakia Ferdausi Khan, and Arpan Maitra collected data. S. M. Sharf‐Ul‐Alam analyzed data and wrote the first draft. Md. Ziaul Islam and Md. Abdullah Saeed Khan critically reviewed the first draft. All authors reviewed and approved the final draft.

## Funding

The authors have nothing to report.

## Disclosure

No patients or the public were involved in the design, or conduct, or reporting, or dissemination plans of our research.

## Conflicts of Interest

The authors declare no conflicts of interest.

## Supporting information




**Supporting File**: puh270163–sup–0001–SuppMat.docx

**Supporting File**: puh270163–sup–0002–SuppMat.docx

## Data Availability

Anonymized data are available upon request from the corresponding author.
