# Supplementary material for: Pathways and Experiences of Children in Beach Vending: Findings From Cox's Bazar Sea Beach in Bangladesh
Source: Public Health Chall. 2025 Nov 27;4(4):e70163. doi: 10.1002/puh2.70163 (PMC12659252; doi:10.1002/puh2.70163)
Supplement: Supplementary file 2 — Supporting File: puh270163–sup–0002–SuppMat.docx [file PUH2-4-e70163-s001.docx]

**Title: Pathways and Experiences of Children in Beach Vending: Findings from Cox's Bazar Sea Beach in Bangladesh**

**Annexure**

**In depth Interview Guide**

Assalamu alaikum / Adab, my name is __________, and I am conducting a study on “Pathways and Experiences of Children in Beach Vending: Findings from Cox's Bazar Sea Beach in Bangladesh”. I would like to ask you some questions about your engagement as a beach vendor and your professional experiences. The information you provide will be kept strictly confidential and used only for research purposes. Your participation is voluntary, and you can stop the interview at any time. The interview session will be recorded with a digital recorder, subject to the permission of the informant. If you agree, please respond to the following questions/statements.

**Section-1: Particulars of the participants**

Id no: Name:

Age (Years): Address:

Contact no (If any):

Date of Interview: Interview time: Start time: End time:

**Section-2: Information on families of the participants**

| **Statements/ Questions** | **Response** |
| --- | --- |
| Please tell me about your background  (Name, age, education, daily routine) |  |
| Would you please tell me about your family?  (Family composition, occupation of members, your position among siblings, health of family members, living conditions including housing, electricity, and other utilities) |  |
| Would you please explain the financial aspect of your family?  (Earning members, source of earnings, sufficiency of earning to meet basic needs, challenges of earning, debt or additional expanses) |  |
| Would you please narrate the relationship of your family with your neighbor and relatives?  (Social and financial interaction, attitude, and behavior of neighbors and relatives towards the family) |  |

**Section-3: Information on pathways to beach vending**

| **Statements/ Questions** | **Response** |
| --- | --- |
| Would you please describe your vending profession?  (Type of vending, preparation/collection of vending materials, place and timing of vending, earnings, assistance from family members and peers) |  |
| Would you please explain the reasons for being engaged in this vending profession?  (Financial reasons, familial and social reasons, forceful engagement) |  |
| How will you evaluate your engagement in vending profession?  (Family survival, extra earning opportunity, meeting extra personal expenses) |  |

**Section-4: Information on experiences of beach vending**

| **Statements/ Questions** | **Response** |
| --- | --- |
| Would you please describe your experiences as a beach vendor?  (Vending experience at the beach, interaction with fellow vendors and customers, working conditions) |  |
| Would you please explain the challenges you faced during beach vending?  (Physical problems, mental challenges, familial and social challenges, academic and daily life interruptions) |  |
| Would you please tell me about your goals and future plans?  (Future aim and dreams, possible contribution for oneself and family, achievements through earning) |  |

Thank you for your sincere participation in the interview.
